# 1-Methyltryptophan Modifies Apoplast Content in Tomato Plants Improving Resistance Against *Pseudomonas syringae*

**DOI:** 10.3389/fmicb.2018.02056

**Published:** 2018-08-31

**Authors:** Loredana Scalschi, Eugenio Llorens, Ana I. González-Hernández, Mercedes Valcárcel, Jordi Gamir, Pilar García-Agustín, Begonya Vicedo, Gemma Camañes

**Affiliations:** ^1^Grupo de Bioquímica y Biotecnología, Área Fisiología Vegetal, Departamento de Ciencias Agrarias y del Medio Natural, Universitat Jaume I, Castellón, Spain; ^2^Unidad Mixta de Investigación Mejora de la Calidad Agroalimentaria UJI-UPV, Departamento de Ciencias Agrarias y del Medio Natural, Universitat Jaume I, Castellón, Spain; ^3^Departamento de Microbiología del Suelo y Sistemas Simbióticos, Estación Experimental del Zaidín, Consejo Superior de Investigaciones Científicas, Granada, Spain

**Keywords:** induced resistance, 1-methyltryptophan, Pseudomonas syringae, *Solanum lycopersicum*, apoplast

## Abstract

Plants can produce numerous natural products, many of which have been shown to confer protection against microbial attack. In this way, we identified 1-methyltryptophan (1-MT), a natural compound produced by tomato plants in response to *Pseudomonas syringae* attack, whose application by soil drench provided protection against this pathogen. In the present work, we have studied the mechanisms underlying this protection. The results demonstrated that 1-MT can be considered a new activator of plant defense responses that acts by inhibiting the stomatal opening produced by coronatine (COR) and could thereby, prevent bacteria entering the mesophyll. Besides, 1-MT acts by blocking the jasmonic acid (JA) pathway that, could avoid manipulation of the salicylic acid (SA) pathway by the bacterium, and thus hinder its growth. Although the concentration of 1-MT reached in the plant did not show antimicrobial effects, we cannot rule out a role for 1-MT acting alone because it affects the expression of the *fliC* gene that is involved in synthesis of the flagellum. These changes would result in reduced bacterium motility and, therefore, infective capacity. The results highlight the effect of a tryptophan derivative on induced resistance in plants.

## Introduction

Plants have developed a variety of chemical and physical basal defense mechanisms to cope with environmental changes and pathogenic invasions. When pathogens penetrate the superficial layer of plant leaves, they encounter early acting post-invasive defense systems. The ability to detect and activate a defense response against potentially pathogenic microorganisms is important for stopping disease progression. The activation of these defense systems is linked to the recognition of pathogen-associated molecular patterns (PAMPs), such as lipopolysaccharides and flagellin in Gram-negative bacteria, or chitin and ergosterol in higher fungi (Nürnberger et al., 2004). Besides, in response to the delivery of pathogen effector proteins, plants produce resistance proteins (R proteins) to either directly or indirectly monitor the presence of the pathogen effector proteins (Chisholm et al., 2006).

The plant–pathogen interaction is a system that depends on the lifestyle of the microorganism and plant species. The pathosystem *Pseudomonas syringae* vs. tomato is a widely used model to study the plant–pathogen interaction: firstly given its genetic tractability and pathogenicity, *Pseudomonas syringae* pv. tomato DC3000 is an appropriate strain used to investigate plant–microbe interactions (Cuppels, 1986; Elizabeth and Bender, 2007; Uppalapati et al., 2008); secondly, tomato is an economically important plant and its study could be extended to other species closely related to it that are important for agriculture.

Plant immunity against *Pseudomonas* involves multiple responses (Xin and He, 2013). Among them, plant hormones play an important role in modulating plant resistance to *P. syringae*. Salicylic acid (SA) acts at different levels of plant defense against biotrophic and hemibiotrophic pathogens and is associated with the induction of *PR* (pathogenesis-related) genes expression (Vlot et al., 2009). Jasmonic acid (JA) is known to protect plants against necrotrophic pathogens and herbivores (Thomma et al., 2001). Although previous works have shown the antagonistic interactions between the SA- and JA-mediated signaling pathways (Niki et al., 1998; Koornneef et al., 2008; Pieterse et al., 2012), synergistic interactions have also been described (Mur et al., 2006; Halim et al., 2009; Scalschi et al., 2013).

Abscisic acid (ABA) is also involved in biotic stress responses in a complex manner. Several authors have shown that ABA promotes *Pseudomonas* infection (Thaler and Bostock, 2004; Cao et al., 2011; Robert-Seilaniantz et al., 2011). Nevertheless, ABA is the hormone responsible for guard cell closure control, which is a very important aspect to prevent the entrance and reproduction of pathogens in the mesophyll (Melotto et al., 2006; Gudesblat et al., 2009b). In relation to this, Fernández-Crespo et al. (2015) have shown that tomato plants grown with NH_4_^+^ as the only source of N display higher basal ABA accumulation and more closed stomata than control plants, which reduce the entry of *Pseudomonas* in the mesophyll apoplast.

*Pseudomonas syringae* pv. tomato DC3000 is a bacterial pathogen with two lifestyles: an initial epiphytic phase on the leaf surface and an endophytic phase in the apoplastic space, which it can access via wounds or natural plant openings, such as stomata (Beattie and Lindow, 1995; Melotto et al., 2008). *P. syringae* survival within the plant depends on several factors, such as its motility and ability to enter the mesophyll, the availability of nutrients in the apoplast, and the ability to cope with host defense systems. *P. syringae* uses flagellar motility to locate at optimal sites for nutrient acquisition or to avoid toxic substances. Therefore, flagella play a role by either stimulating host defense or in disease causation (Xin and He, 2013). This bacterium also possesses many virulence factors, like proteinaceous effectors, which are secreted through the type III secretion system directly into host cells, and a polyketide phytotoxin called coronatine (COR), which structurally mimics plant hormone jasmonate isoleucine and triggers the activation of JA-dependent defense responses to lead to the suppression of SA-dependent defense responses (Laurie-Berry et al., 2006).

*Pseudomonas syringae* develops its pathogenic phase in the apoplast. The apoplastic space is the plant cell compartment outside the cell membrane, where the first interaction between plants and pathogens takes place. It is an environment that is acidic, low in nitrogen, and rich in plant-derived sugars, such as fructose and glucose (Rico and Preston, 2008). It is known that both abiotic and biotic stresses are able to change its content. Therefore the apoplast can be considered one of the primary lines of defense against pathogen invasion because it may contain antimicrobial molecules (Bednarek et al., 2010) and reactive oxygen species (Torres et al., 2006; Torres, 2010), which might directly affect the pathogen or serve as plant response inducers. For this reason, an analysis of the changes that occur in the apoplast during pathogen infection and in plant-induced defense is important to gain a better understanding of the plant–pathogen interaction and disease control in early development stages.

An alternative system to use pesticides in agriculture is to induce plant defense against pathogens by treatments with natural compounds. The search for such compounds that are effective in protecting crops against pathogens allowed us to determine the effectiveness of hexanoic acid (Hx). Hx acts as an inducer of plant defenses by means of a priming mechanism against pathogens with different lifestyles (Vicedo et al., 2009; Llorens et al., 2013; Scalschi et al., 2013). In order to characterize the priming mechanism conferred by Hx, a metabolic profile of tomato plants infected by *Botrytis cinerea* or *P. syringae*, and treated with Hx, has been performed (Camañes et al., 2015). In that study, the compound 1-methyltryptophan (1-MT) was detected and its presence was associated with tomato-*P. syringae* and tomato–*B. cinerea* interactions, and with Hx-induced resistance. Moreover, the same work demonstrated that root application of 1-MT significantly reduces the infection of both pathogens. In recent years, other tryptophan-derivates have emerged as relevant defene mechanisms, which have conferred resistance to the necrotrophic fungus *Plectosphaerella cucumerina* in *Arabidopsis thaliana* (Sanchez-Vallet et al., 2010; Gamir et al., 2012).

The aim of this work was to study the modifications produced by 1-MT in the plant that improve its resistance against the pathogen and its possible direct effect on the bacteria. For this purpose, we treated tomato plants with 1-MT by soil drenching. Then the metabolic and transcriptomic profiles were analyzed in the plant and apoplast extracts. We also analyzed the transcriptomic changes in the genes related to the pathogenicity and virulence of bacteria.

## Materials and Methods

### Microbial Strains, Growth Conditions, and Plant Material

*Pseudomonas syringae* pv. tomato strains used in the present study were DC3000 and 775EGFP (DC3000 labeled with GFP, Río-Álvarez et al., 2014). Rifampicin and kanamycin, respectively, were added to King B medium (KB) at 50 μg mL^-1^. Tomato seeds (*Solanum lycopersicum* Mill. cv. Ailsa Craig) were germinated in vermiculite in a growth chamber under the following environmental conditions: light/dark cycle of 16/8 h, temperature of 24/18°C, light intensity of 200 μmol m^-2^ s^-1^, and 60% relative humidity. Seeds were irrigated with distilled water for a week and the next 3 weeks with Hoagland solution (Hoagland and Arnon, 1950). The pH of the nutrient solution was adjusted to 5.8–6.0 with 1 mM KOH.

### *P. syringae* Bioassays

Four-week-old tomato plants were treated with nutrient solution or 20 mL of 1-MT (5 mM) at pH = 6 dissolved in the nutrient solution 72 h before inoculation. *P. syringae* pv. tomato DC3000 was grown in KingB (KB) medium at 28°C for 24 h. Bacterial suspensions were adjusted to 5 × 10^5^ cfu mL^-1^ in sterile MgSO_4_ (10 mM) with 0.01% of Silwet L-77 surfactant (Osi Specialties, Danbury, CT, United States). Tomato plants were challenged by dipping with *P. syringae* and the disease rate was scored as described by Camañes et al. (2015). The third and fourth leaves of 10 plants for every treatment were sampled at different time points [6, 24, and 48 h post-inoculation (hpi)] and frozen at -80°C.

### Chromatographic Analysis

Leaves were frozen in liquid N_2_, ground, and lyophilized. For hormonal analysis, dry tissue (0.05 g) was homogenized in 2.5 mL of ultrapure water and 100 ng mL^-1^ of internal standards (*d^6^-*ABA, *d^4^-*SA and dihydrojasmonic acid) was added. The samples were centrifuged at 5,000 rpm for 45 min at 4°C. The supernatant was partitioned against diethylether, dried in a speed vacuum and resuspended in 90:10 H_2_O:MeOH. For amino acids and 1-MT analysis, dry tissue (0.1 g) was homogenized with 750 μl of extraction solution composed by: 80 μl of distilled water, 200 μl of chloroform and 470 μl of methanol per sample. Moreover, a mixture of internal standards was added prior to extraction (100 ng of Phe ^13^C_9_^15^N and 100 ng of Thr ^13^C_4_^15^N). In both cases, a 20 μl aliquot was injected into an Acquity ultra-performance liquid chromatography system (UPLC) with an ACQUITY UPLC BEH C18 column (1.7 μm 2.1 × 50 mm) (Waters, Mildford, MA, United States), which was interfaced with a triple quadrupole mass spectrometer (TQD, Waters, Manchester, United Kingdom). The solvent gradient used was 95% H_2_O: 5% MeOH: 0.1% CHOOH to 5% H_2_O: 95% MEOH: 0.1% CHOOH over 8 min. The MASSLYNX NT software version 4.1 (Micromass^1^) was used to process the quantitative data from calibration standards and plant samples.

Hormonal, amino acids, and 1-MT analyses were also performed for the apoplast extracted at 48 hpi from control and treated plants, infected and non-infected. 100 ng mL^-1^ of internal standards were directly added to the apoplast after extraction and further processed with the UPLC as described above.

### Analysis of Gene Expression by Quantitative Real-Time Polymerase Chain Reaction (qRT-PCR)

RNA was extracted from frozen tomato leaves using the E.Z.N.A Plant RNA Kit^2^ according to the manufacturer’s instructions. Leaf tissue from 10 treated and untreated plants were collected at the specified time points post-inoculation. A total of 1 μg of total RNA was digested using 1 U of RNase-free DNase (Thermo Fisher^3^) and incubated for 30 min at 37°C. For each sample, cDNA was synthesized using 1 μg of total RNA in 10 μL total reaction volume with oligo dT primer and primescript RT enzyme mix 1 (Primescript RT reagent kit, TaKaRa^4^). Forward and reverse primers (10 μM) were added to 5 μl of Maxima SYBR Green/ROX qPCR Master Mix (Thermo Fisher), as well as 1 μl of diluted cDNA and Milli-Q sterile water up to a total reaction volume of 10 μl. Quantitative PCR was carried out using the StepOne^TM^ Real-Time PCR System (see footnote 3). A list of the primers used for the quantitative Real Time-PCR is shown in **Supplementary Table S3**. Levels of *EF1α* gene expression were used as an internal housekeeping control. The amplification efficiency for each primer pair was calculated using serial cDNA dilutions. Differences in cycle numbers during the linear amplification phase between samples from treated and untreated plants were used to determine differential gene expression.

To extract bacterial RNA from infected plants we used a protocol for extracting RNA from *P. syringae* recovered from infected leaves as described by Yu et al. (2013). Briefly, for isolation of RNA, the Qiagen RNeasy Bacteria Mini Kit^5^ was used according to manufacturer’s instructions. Reverse transcription was performed in 10 microL total reaction volume with random hexamers and primescript RT enzyme Mix 1 (Primescript RT reagent kit, TaKaRa). Quantitative PCR were carried out as described above. Primers used for the assay are described in **Supplementary Table S2**. Relative levels of the monitored genes were normalized with *recA* that was used as an internal reference.

### Apoplast Extraction

Apoplast extraction was carried out 48 h after *Pseudomonas syringae* inoculation by using the infiltration-centrifugation method as described by O’Leary et al. (2014). Leaflets of the third and fourth true leaves of 10 one-month-old plants were used for each treatment. Briefly, this technique is a two-step method that essentially involves replacement of the apoplastic air space with sterile distilled water, which mixes with the native apoplastic fluid, followed by recovery of the infiltration/apoplastic mixture by gentle centrifugation of the leaves. The cytoplasmic contamination of apoplast was estimated as is described in Rico and Preston (2008). Prior to subsequent analyzes, the apoplast extract was diluted twice in distilled water and filtered on a cellulose syringe filter pore size 0.2 μm, in order to avoid bacterial contamination. Four biological replicates of apoplast extracted from control and treated plants, infected and non-infected were performed.

### Stomatal Aperture Analysis

Tomato plants were maintained in the same culture conditions and treated as described for the *P. syringae* bioassays. The third and fourth leaves were collected and placed on glass slides with the adaxial epidermis in contact with dental resin (Geisler et al., 2000; Delgado et al., 2011). Stomatal aperture analysis was performed as described by Scalschi et al. (2013).

### Analysis of Bacteria Presence in the Leaves With Confocal Microscopy

For confocal microscopy analysis, *P. syringae* pv. tomato 775EGFP strain, transformed with pUFZ15 containing the GFP fluorescent protein (Río-Álvarez et al., 2014) was used. In order to check 775EGFP strain response to 1-MT treatment, bacteria were inoculated as described above. Results showed a similar effect as for DC3000 strain (data not shown). For the confocal analysis, inoculated tomato leaves from treated and non-treated plants were examined at different time periods in order to check for the presence of the bacteria on the surface and in the mesophyll. The GFP signal and chlorophyll autofluorescence were collected on an Inverted Confocal Microscope Leica TCS SP8 (Leica Microsystems, Wetzlar, Germany) using 488 nm ray line of the argon laser for their excitation. GFP fluorescence was collected between 500 and 540 nm by a HyD detector while the fluorescence emitted by the chlorophyll was collected between 650 and 700 nm by a PMT detector. The same gain and offset settings were used for the different treatments. The images were processed using the LAS X (Leica Microsystems). Five leaves were observed for each treatment and for each time point.

### Determination of Sugar Concentration

Fructose, glucose, and sucrose concentration was determined following the method described by Cebolla-Cornejo et al. (2012) with some modifications using an Agilent 7100 capillary electrophoresis system (Agilent Technologies, Waldbronn, Germany). Prior to use, uncoated fused silica capillaries (67 cm total length, 60 cm effective length, 375 μm outside diameter, 50 μm internal diameter) from Polymicro Technologies (Phoenix, AZ, United States) were conditioned at 50°C with NaOH 1 N (5 min), NaOH 0.1 N, and MilliQ water (10 min). Before each working session, the capillary was rinsed for 30 min with the running buffer (20 mM PDC and 0.1% w/v HDM at pH 12.1). Between runs, the capillary was flushed with 60 mM SDS (3 min), MilliQ water (1 min) and the running buffer (2 min). Samples were diluted ½ with MilliQ water, filtered (0.2 μm) and then injected hydrodynamically at 6900 Pa during 30 s. Separations were performed at -25 kV and 20°C, with indirect detection at 214 nm.

### *In vitro* Bacterial Growth Assay

We tested 1-MT effect against *P. syringae* pv. tomato DC3000 in LB medium to which, the compound was added at a final concentration of 0.5, 1, 2.5, and 5 mM. 1-MT was prepared in distilled water adjusted to pH = 6, and sterilized by filtration. 2xLB was prepared and diluted with 1-MT and water until reaching the concentration of use. The inoculum was obtained as described above. The growth assay was carried in a microtiter plate, in a total volume of 250 μl LB with or without 1-MT, using an initial bacterial concentration of about 5 × 10^5^ cfu mL^-1^. Bacterial growth was monitored by measuring optical density in a microplate reader (MB-580, Heales) at 20 hpi in the medium. Eight independent replicates were performed for each condition.

Bacterial growth assays were also performed in extracted apoplast from treated or untreated plants, infected or non-infected using an initial bacterial concentration of about 1 × 10^6^ cfu mL^-1^.

### Swimming Assays

To analyze the effect of the treatment on the mobility of the bacteria *in vitro, P. syringae* inoculum was obtained as described above. Five microliters of the bacterial suspension were inoculated onto KB agar plates (containing 50% KB and 0.25% agar) with or without 1-MT at 1.5 or 5 mM, with a sterile pipette tip. The plates were incubated at 28°C. Five plates by treatments were used. The diameter of the culture was measured at 72 hpi.

### Statistical Analysis

All experiments were conducted at least three times. Data from different repetitions was analyzed together due to the fact that analysis of variance (ANOVA) did not show significant differences (*p* < 0.05) between repetitions in each experiment. All data of this study were tested for normality, homogeneity of variances, and residual patterns. When ANOVA showed significant differences between variables, mean values were compared using the Fisher’s least significant difference (LSD) test at the 95% confidence. Statistical analyses were performed using the software Statgraphics Centurion XVI (Statpoint Technologies, Warrenton, VA, United States). Heatmaps were generated with the levels of aminoacids, hormones and sugars following log transformation using the gplots package of R statistical program (version 3.4.3).

## Results

We have previously reported that 1-MT protects tomato plants against *P. syringae* and *B. cinerea* (Camañes et al., 2015). The present paper examined the protection of tomato plants by 1-MT against *P. syringae.* The results obtained were similar to those described previously by Camañes et al. (2015); we observed an average reduction of approximately 40% in disease symptoms and an average drop of 80% in the number of colony-forming units (cfu) after treatment. Therefore, we extended our analyses to study the mechanisms underlying the resistance against *P. syringae* conferred by 1-MT. For this purpose, metabolic and transcriptomic analyses of leaf samples were performed in tomato plants.

### Changes in the Hormonal Pattern of the 1-MT-Treated Tomato Plant Upon *P. syringae* Infection

To further confirm the possible role of the different signaling pathways in 1-MT-induced resistance, we analyzed the hormonal levels in the control and the 1-MT-treated tomato plants at three time points after *P. syringae* infection.

**FIGURE 1:**
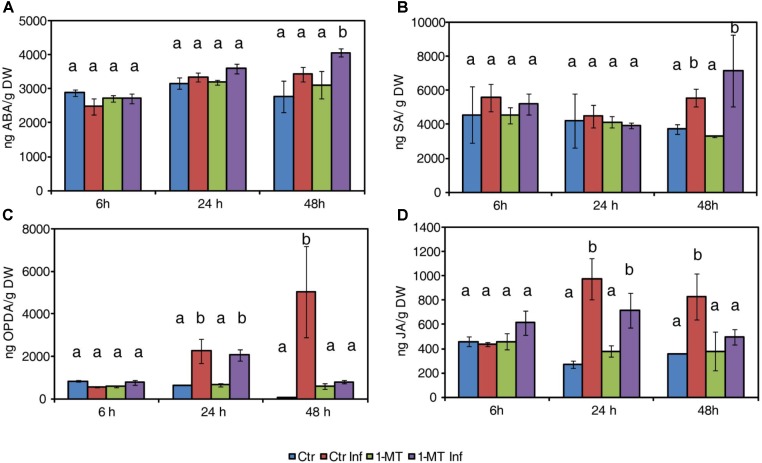
Hormone levels in water- and 1-MT-treated tomato plants on *P. syringae* infection. Leaves were collected at 6, 24, and 48 hpi and ABA **(A)**, SA **(B)**, OPDA **(C)**, and JA **(D)** levels were determined by ultra-performance liquid chromatography (UPLC)-mass spectrometry. Data show the average of three independent experiments of a pool of 10 plants per experiment ± SE. Different letters indicate statistically significant differences between treatments at the same time point (*p* < 0.05; least-significant difference test). Ctr, untreated and uninfected plants; Ctr Inf, untreated and inoculated plants; 1-MT, treated plants; 1-MT Inf, treated and inoculated plants.

Abscisic acid levels significantly increased at 48 hpi in the treated and infected plants compared with the untreated infected plants (**Figure 1A**). Besides ABA, SA levels also increased in the treated and infected plants at this time point (**Figure 1B**) but, no significant differences were observed compared with the untreated infected plants. While oxylipin 12-oxo-phytodienoic acid (OPDA) and JA increased in untreated and treated infected plants at 24 hpi, this increase was observed only in the untreated infected plants at 48 hpi (**Figures 1C,D**). These results indicate that 1-MT could block the oxylipin pathway.

### 1-MT Induced the Expression of Genes Involved in Defense Pathways

As 1-MT treatment changed the hormonal profile, we next examined the expression patterns of marker genes for the ABA (*ASR1*), SA (*PR1* and *PR5*), and JA (*AOC*) signaling pathways in the leaf samples taken from the treated and untreated plants at 6, 24, and 48 hpi.

**FIGURE 2:**
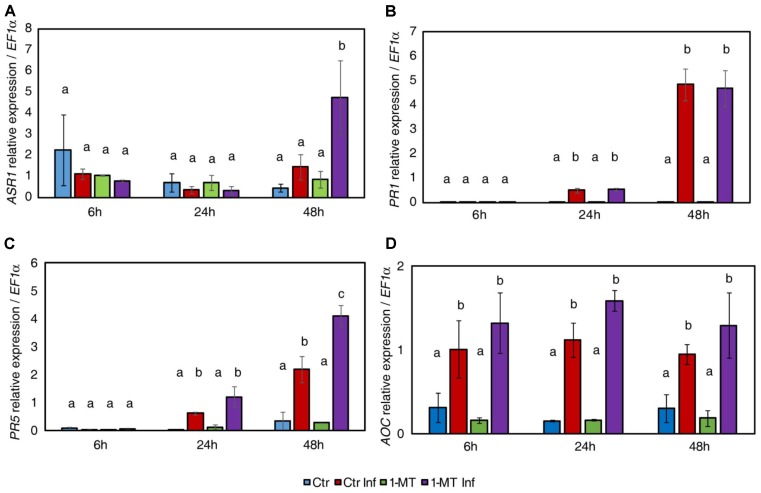
Gene expression profile of plant defense pathways in water- and 1-MT-treated tomato plants on *P. syringae* infection. Leaves were collected at 6, 24, and 48 hpi and expression levels of marker genes of ABA (*ASR1*) **(A)**, SA (*PR1* and *PR5*) **(B,C)**, and JA (*AOC*) **(D)** signaling pathways were analyzed. The results were normalized to the *EF1α* gene expression measured in the same samples. Data show the average of three independent experiments of a pool of 10 plants per experiment ± SE. Statistical analysis was carried out between samples collected at the same time point. Different letters indicate statistically significant differences between treatments (*p* < 0.05; least-significant difference test). Ctr, untreated and uninfected plants; Ctr Inf, untreated and inoculated plants; 1-MT, treated plants; 1-MT Inf, treated and inoculated plants.

The results show that 1-MT increased *ASR1* expression at 48 hpi (**Figure 2A**). This correlated with the accumulation of ABA observed at this time point and suggests that the ABA pathway might play a role in 1-MT-induced resistance. Regarding the SA pathway, no differences in the expression levels of *PR1* were observed between the treated and untreated plants upon infection, consistent with the hormonal analysis (**Figure 2B**). However, *PR5* expression was induced by treatment at 48 hpi (**Figure 2C**). This result indicates a possible role for this protein in 1-MT-mediated protection. Even though OPDA and JA levels were lower in the treated and infected plants, no differences in the expression levels of the marker genes of the oxylipin pathway were observed in the treated *versus* the untreated plants upon infection (**Figure 2D**).

### 1-MT Inhibits Stomatal Opening and Reduces Bacterial Mesophilic Colonization

**FIGURE 3:**
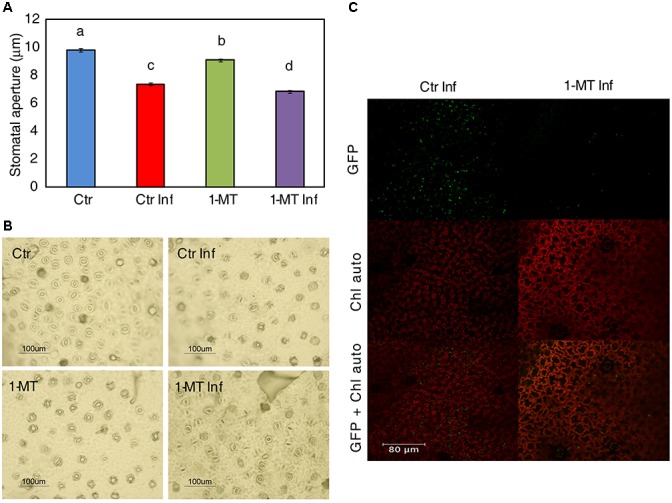
1-MT-treatment affects stomatal opening. **(A)** Stomatal apertures were analyzed ‘*in situ*’ in leaflets of water (Ctr) and 1-MT treated tomato plants at 24 hpi. Results are average ± SE (*n* < 50 stomata). Different letters represent statistically significant differences (*p* < 0.05; least-significant difference test). **(B)** Representative photographs of stomatal aperture taken after 1-MT treatment. **(C)** Confocal images showing a low number of bacteria in 1-MT treated plants and colonies formed by a greater number of cells in non-treated plants 24 hpi.

Abscisic acid is known to play a key role in regulating stomatal closure. In this context, the increase in ABA level in plants treated and inoculated, indicated that 1-MT treatment may act by blocking the re-opening of stomata promoted by COR. Therefore, the effect of 1-MT treatment on stomatal opening was studied. The results showed that both the treated and untreated infected plants had more closed stomata than the uninfected ones in the early hours of infection (6 hpi, data not shown). Nevertheless, the stomata of the treated and infected plants remained more closed at 24 h; this could affect bacterium entry and establishment in the mesophyll. These results, together with those observed for the induction of ABA and the ABA marker gene, support the idea that 1-MT treatment might regulate stomatal closure and make plant defense more effective (**Figures 3A,B**).

To study how the bacterium colonized the mesophyll, confocal microscopy studies were conducted using a *P. syringae* pv tomato DC3000 strain labeled with GFP (Río-Álvarez et al., 2014). Pictures taken at 3, 24, and 72 hpi showed that although bacteria were observed on the leaf surface at 3 hpi, at 24 h their presence on the leaf surface decreased in both the treated and untreated plants. Moreover, at 24 hpi, small groups of bacteria began to appear in the intercellular space in the control plants while only isolated bacterial cells were seen in the treated plants (**Figure 3C**).

### Apoplastic Changes Induced by 1-MT Affect *P. syringae* Survival *in vitro*

**FIGURE 4:**
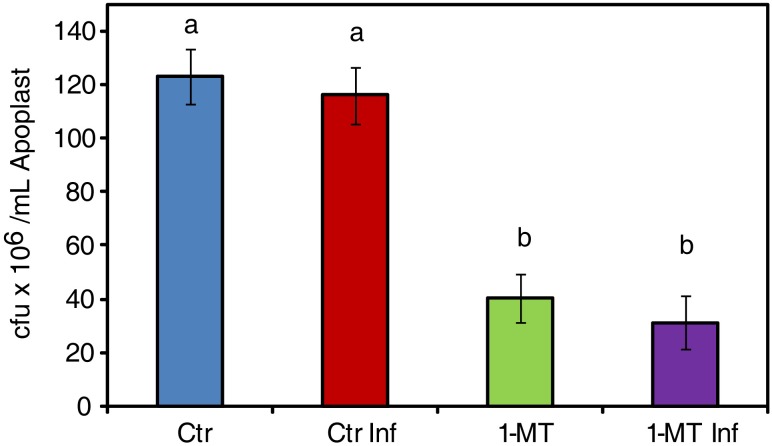
Survival of *P. syringae* in apoplast extract. 10^6^ cfu mL^-1^ of bacteria initial concentration was maintained in apoplast extract from uninfected (Ctr) infected (Ctr Inf) treated (1-MT) and treated and infected (1-MT Inf) plants. Bacterial growth was measured at 24 h. Data show the average of eight samples per condition of an experiment ± SE. The experiment was repeated three times with similar results. Different letters indicate statistically significant differences (*p* < 0.05, least-significant difference test).

The endophytic phase of bacteria takes place in the apoplast so, apoplast composition might be another factor affecting bacterial colonization of the mesophyll. To study this hypothesis, apoplast extraction was performed 48 hpi from both the treated and untreated plants, as previously described by Rico and Preston (2008). To assess the apoplast’s capacity to inhibit bacterial growth, a bacterial suspension of 10^6^ cfu mL^-1^ was grown on the extracted apoplast and its development was monitored for 24 hpi. The results showed significantly less growth for the bacterial populations inoculated on the apoplast extracted from the treated plants than in the apoplast extracted from the untreated plants (**Figure 4**). However, no differences in bacterial growth were found when using apoplast extracted from treated plants with or without infection. Therefore, we conclude that the changes in the leaf apoplast caused by 1-MT treatment affected the survival of bacteria independently of infection.

### Changes in the Hormonal Pattern, Sugar, and Amino Acid Content in the Apoplast Upon *P. syringae* Infection

**FIGURE 5:**
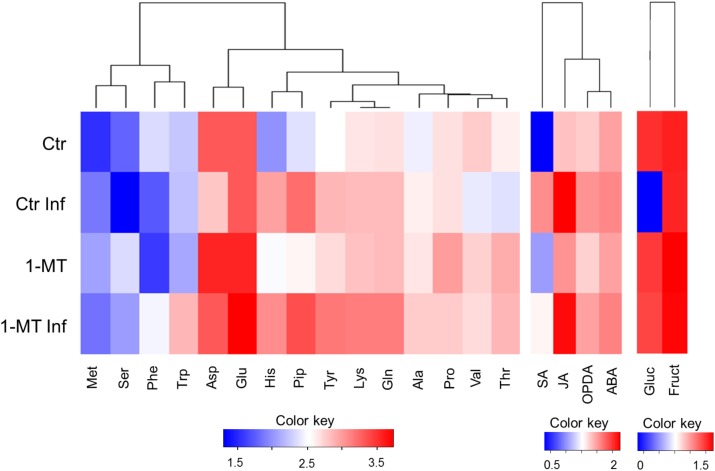
Heatmap visualization of the content of metabolites in the apoplast in water- and 1-MT-treated tomato plants on *P. syringae* infection. Apoplast extract was collected at 48 hpi and hormone, amino acid and sugar content were analyzed. Ctr, untreated and uninfected plants; Ctr Inf, untreated and inoculated plants; 1-MT, treated plants; 1-MT Inf, treated and inoculated plants. Columns present the different metabolites analyzed; Color key indicates log transformation of metabolite concentration, blue: Lowest, red: highest.

Having observed the ability of the apoplast to inhibit bacterial growth, we compared the metabolic responses between the apoplast of the treated and untreated plants, with or without infection (**Figure 5** and **Supplementary Table S1**). First, we analyzed the levels of the plant hormones involved in plant defense processes. This showed that the hormone levels in the apoplast were much lower than those detected inside cells. Moreover, the levels of most of the analyzed hormones were significantly higher in the infected plants than in the uninfected ones, regardless of treatment. This suggests that elevated hormone levels are associated with the presence of the pathogen. Therefore, it appears that no studied hormone itself was able to exert an effect on the control of bacterial growth in the apoplast. The amino acid content was also analyzed, and the results showed that threonine (Thr) levels were higher in the infected and uninfected treated plants. After infection, Thr levels lowered in the untreated plants; tryptophan (Trp) levels were higher only in the treated and infected plants. No significant differences were found between the treated and untreated plants for the other amino acids analyzed.

Finally, sucrose, fructose, and glucose levels were determined. While glucose and fructose were detected in all the experiments, sometimes at very low levels, no sucrose was detected in any experiment. Glucose was not observed in the apoplast extracted from the infected control plants, in which higher bacterial populations were found. This sugar was probably not detected because it was metabolized by bacteria. This is consistent with the amounts of glucose detected in the apoplast of treated and infected plants that, appeared to be inversely correlated with the amounts of bacteria. Interestingly, the absence of glucose did not negatively affect the growth of the bacteria inoculated in the apoplast extracted from the untreated and infected plants (**Figure 4**). Furthermore, the growth of these bacteria was similar to that of the bacteria inoculated on the apoplast extracted from the control plants, and was higher than the growth of the bacteria inoculated on the apoplast of the treated and treated and infected plants. Fructose levels were also higher in the apoplast of the infected and uninfected treated plants, but no significant differences were observed compared with the untreated plants.

### *P. syringae* Growth Is Dependent on 1-MT Concentration

**FIGURE 6:**
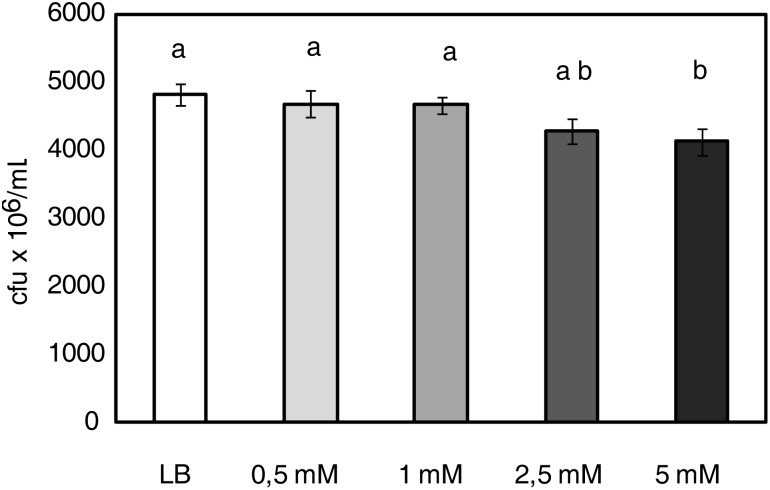
*P. syringae* growth is dependent on 1-MT concentration. 5 × 10^5^ cfu mL^-1^ of bacterial initial concentration was grown in LB or LB supplemented with 0.5, 1, 2.5, and 5 mM 1-MT. Bacterial growth was monitored by measuring optical density in a microplate reader (at 20 h after inoculation in the medium). Data show the average of eight samples per condition of an experiment ± SE. The experiment was repeated three times with similar results. Different letters indicate statistically significant differences (*p* < 0.05, least-significant difference test).

To test the direct effect of 1-MT against *P. syringae*, the growth of the bacterium was examined in both the absence and presence of different 1-MT concentrations (0.5, 1, 2.5, and 5 mM) in LB medium. The results showed that of all the tested concentrations, only 5 mM 1-MT produced a 15% inhibition of bacterial growth; 1-MT at 0.5, 1, and 2.5 mM concentrations did not reduce the growth of the bacteria compared with the control (**Figure 6**).

As the root treatment concentration (5 mM) affected bacterial growth, the content of this compound in leaves and the apoplast was analyzed. The results (**Table 1**) showed that the concentration in both the apoplast and plant did not exceed 2 mM and was, therefore, below the concentration that could affect bacterial growth.

**Table 1:** 1-MT concentration determined in leaves or in apoplast.

***In planta***	Control	n.d.
	Infected plant	n.d.
	1-MT	1.8 ± 0.5^a^ mM
	1-MT infected plants	1.2 ± 0.2^a^ mM
**Apoplast**	Control	n.d.
	Infected plant	n.d.
	1-MT	0.007 ± 0.03^b^ mM
	1-MT infected plants	0.008 ± 0.03^b^ mM

1-MT concentration was determined by UPLC-MS^1^ in leaves of tomato plants or in apoplast extract in Ctr, untreated and uninfected plants; Ctr Inf, untreated and inoculated plants; 1-MT, treated plants; 1-MT Inf, treated and inoculated plants. Data show the average of three independent experiments ± SE. Different letters indicate statistically significant differences (*p* < 0.05, least-significant difference test). n.d., no detected.

### 1-MT Affects *P. syringae* Motility

**FIGURE 7:**
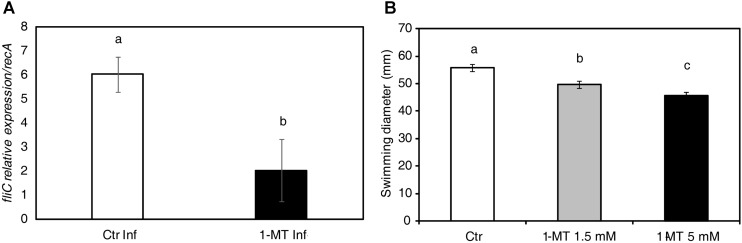
1-MT affects the motility of *P. syringae.* **(A)** RNA extraction was performed from bacteria extracted from treated and untreated plants 72 hpi. Relative expression of *fliC* during bacterial growth was analyzed. The *recA* gene was used as an endogenous reference gene. **(B)** Swimming diameter was determined 72 hpi in KB agar plates (containing 50% KB and 0.25% agar) with or without 1-MT at 1.5 or 5 mM where 5 μl of 5 × 10^5^ cfu mL^-1^ bacterial suspension was inoculated. The plates were incubated at 28°C and five plates by treatment were measured. The results represent the average of three independent experiments ± SE. Different letters indicate statistically significant differences (*p* < 0.05, least-significant difference test).

To test whether bacterial virulence was altered by 1-MT treatment, we analyzed the expression of genes related to the pathogenesis and survival of *P. syringae* in the bacteria extracted from both the treated and untreated plants at 72 hpi. The selected genes and their functions are shown in **Supplementary Table S2**. The results indicated that only *fliC* expression, which encodes flagellin, was significantly lower in the bacteria extracted from the 1-MT-treated plants than in the bacteria extracted from the untreated plants (**Figure 7A**). No differences were observed in the other analyzed genes (**Supplementary Figure S1**). These results suggest that 1-MT might affect the motility of bacteria by disrupting flagellum synthesis or function.

Given that *fliC* expression seemed to be affected by 1-MT treatment, we assayed *P. syringae* swimming motility at 72 hpi (**Figure 7B**). The swimming motility of the bacterium was reduced in the cells incubated on plates containing 1.5 and 5 mM of 1-MT compared with the control cells. A more marked reduction in motility was seen at higher concentrations of 1-MT suggesting that *P. syringae* swimming motility in the *in vitro* assays was affected by 1-MT in a concentration-dependent manner. These results confirm our hypothesis that 1-MT affects the synthesis of flagellar components.

## Discussion

In a previous study by our group, characterization of tomato plant response to Hx treatment using a metabolomic approach allowed us to identify 1-MT as one molecule that might be involved in defense against *P. syringae*. Its protective effect has also been demonstrated (Camañes et al., 2015). In the present study, we investigated the mode of action of 1-MT in tomato plants against *P. syringae* by analyzing both the changes occurring in the plant and the bacterial response after treatment.

The importance of SA in defense against *P. syringae* in tomato has been well established. This pathway plays a key role in systemic acquired resistance (SAR) signaling, the synthesis of resistance proteins (PR) (Van Loon, 1997) and the regulation of defense responses, such as stomatal closure (Alvarez, 2000; Melotto et al., 2008). Antagonistic cross-talk is known to exist between SA and JA that, consists of inhibition of SA-dependent responses by the presence of JA (Pieterse et al., 2009). Bacterial virulence factor COR stimulates the JA pathway by suppressing the SA pathway in Arabidopsis and tomato (Brooks et al., 2005; Uppalapati et al., 2005). In this work, we observed that treatment with 1-MT reduced the oxylipin pathway after infection. This reduction could avoid manipulation of the SA pathway by bacteria by allowing the plant to induce the SA response through *PR5*. Although SA and *PR1* expression levels did not significantly differ between the treated and untreated infected plants, the observed levels would have sufficed to enhance the pathogen recognition mechanism by the plant cell (Katagiri et al., 2002). Previous studies have shown that in addition to responding to SA, *PR5* can also respond to either ethylene or MeJA (Reymond and Farmer, 1998). It should be noted that SA is not only a major signal transducer following the recognition of pathogen attack, but that it can also enhance pathogen recognition sensitivity at low levels (Shirasu et al., 1997). One role played by SA in gene-for-gene resistance may be to enhance the recognition mechanism. In this way, only those gene-for-gene interactions with relatively low sensitivities might be strongly affected by a lower SA level (Katagiri et al., 2002). It is also known that besides SA, other hormones may be involved in defense against *P. syringae*, although their role is not entirely clear (Gimenez-Ibanez and Rathjen, 2010). In fact, ABA signaling could play a role in the protection against *P. syringae* mediated by 1-MT because the 1-MT-treated and infected plants displayed higher ABA accumulation and the induction of the *ASR1* marker gene at 48 hpi. Previous studies have uncovered ABA as an important regulator of plant defense responses that, can function positively or negatively depending on the analyzed plant–pathogen interaction (Ton et al., 2009). Specifically, ABA has been found to be a key regulator of pathogen-mediated stomatal closure (Melotto et al., 2008). One *P. syringae* pathogenesis mechanism is stomatal reopening activation mediated by the effector COR that, allows bacteria to enter the mesophyll (Melotto et al., 2006). The ability of bacteria to successfully colonize the apoplastic space is crucial for successful infection. This colonization depends on the ability of bacteria to first access the apoplast and then to survive and reproduce once inside. As for the first requirement to successfully carry out colonization, it is known that stomata effectively function as part of the plant innate immunity (Melotto et al., 2008). In the present work, we observed that the 1-MT treated and infected plants displayed more closed stomata than the control plants, probably because of greater ABA accumulation. This effect of 1-MT treatment on stomatal opening could hinder bacteria entering the mesophyll and, therefore, reduce disease symptoms. Several elicitors and resistance inducers, such as chitosan and Hx have been, respectively, reported to trigger stomatal closure and, therefore, contribute to disease control (Klusener et al., 2002; Gudesblat et al., 2009a; Scalschi et al., 2013).

The second requirement for infection to occur is for bacteria to establish in the apoplastic space. This depends on several factors, such as the ability of bacteria to tolerate preformed defense molecules, to import and metabolize available nutrients, and, ultimately, to express pathogenicity and virulence factors that modulate host defenses and host metabolism by inducing the release of nutrients and water from inside plant cells. Once inside the apoplast, bacteria consume the nutrients present within it and through virulence factors, suppress or evade plant defense molecules (Katagiri et al., 2002, Rico and Preston, 2008). When monitoring the distribution of bacteria over time in the mesophyll of treated and untreated plants using, confocal microscopy we observed how a very small number of bacteria invaded the intercellular space of the treated plants. This could indicate not only more closed stomata, but also reduced bacterial reproduction.

Moreover, the inability of bacteria to colonize the leaf apoplast of the 1-MT-treated plants could also be caused by the presence of antimicrobial factors in the apoplast (Katagiri et al., 2002), a hypothesis that is supported by bacterial growth *in vitro* being lower in the apoplast extracted from the treated plants. This evidence led us to conduct a metabolomic analysis of the apoplast and allowed us to identify differences between the concentration of glucose and several amino acids in the treated and untreated plants. Nevertheless, the lower glucose levels found in the infected plants did not affect the growth of the bacterium *in vitro*; this might indicate that the higher glucose concentrations detected in the treated plants could be associated with increasing plant defense, consistent with other studies (Rojas et al., 2014). Regarding amino acid content, we highlight an increase of Thr in the treated and infected plants, that could positively affect plant protection as has previously been noted for other biotrophs (Stuttmann et al., 2011). Moreover, the levels of Trp, which is considered to be a modulator of defense (Ward et al., 2010), were clearly higher in the treated plants. As previously described (Dominguez and Carrari, 2015), higher Thr and Trp levels could be a consequence of the observed increase in *ASR1* expression in the treated plants. As expected, 1-MT levels were also higher in the treated plants. We, therefore, tested the direct effect of 1-MT on the bacterium. For this purpose, an *in vitro* study was carried out that demonstrated that 5 mM 1-MT applied to roots inhibited cell division; no effect was observed at lower concentrations similar to those detected in leaves and the apoplast. These findings demonstrate that the concentrations of 1-MT observed in the plants were much lower than those found to affect bacterial growth.

Therefore, having ruled out a bactericidal effect of 1-MT at the concentration present in the plant, the possible effect of treatment on the genes involved in the pathogenicity of the bacterium was analyzed because another important factor for the successful colonization of bacteria is their ability to synthesize virulence factors (Rico and Preston, 2008). The analysis of the expression of these genes indicated that treatment did not seem to affect the virulence of bacteria, but did affect their motility through altered *fliC* expression. Expression of *fliC* which is involved in the synthesis of flagellin that is one of the main components of the bacterial flagellum, was low in the treated plants. These results indicate a malfunction of the flagellum that, was confirmed by swimming assays in a semisolid medium in the absence or presence of 1-MT. The motility of bacteria is essential for both their entry and establishment in the mesophyll apoplast and for their capacity to move inside it. Previous works have shown that the flagella of bacteria are also involved in other functions, like forming a secretory system (Young et al., 1999; Haiko and Westerlund-Wikström, 2013). Therefore, their absence could hinder the colonization of the apoplastic space by bacteria.

**FIGURE 8:**
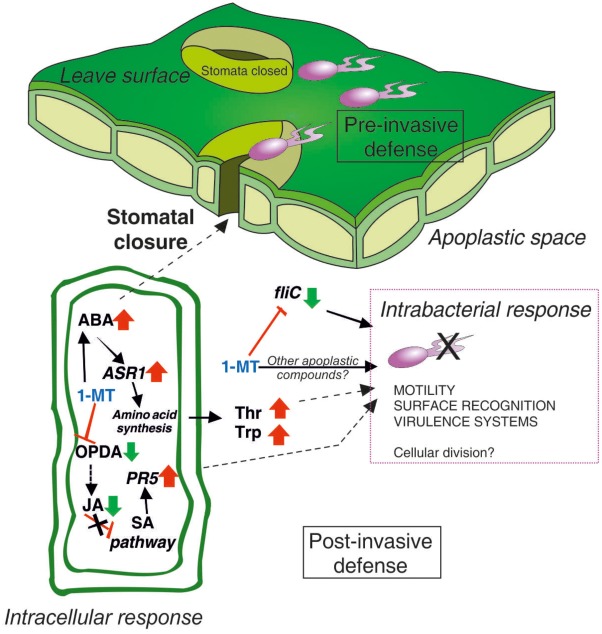
The involvement of 1-MT in tomato–*Pseudomonas syringae* pathosystem. ABA plays a positive role in pre-invasive stomatal immunity by induction of stomatal closure to prevent pathogen entry. 1-MT blocks JA pathway activating this way SA pathway through the expression of *PR5*. The increase in the concentration of Thr and Trp together with 1-MT could be affecting the cellular division of the bacteria. In addition, 1-MT reduces the expression of *flic* gene involved in the synthesis of the flagellum, causing a reduction in the motility of the bacteria and therefore a reduction in its capacity of infection.

A model of the mode of 1-MT action based on the results obtained herein is provided in **Figure 8**. We conclude that the effectiveness of 1-MT treatment can be caused by, in part, the inhibition of stomatal opening regulated by COR as the treated plants showed higher ABA levels and more closed stomata that, could prevent, or at least hinder, the entry of bacteria to the mesophyll, a crucial step for successful colonization. Furthermore, 1-MT appeared to act by blocking the JA pathway that, could avoid the manipulation of SA pathway by the bacterium. Moreover, the entry of bacteria into the mesophyll could also be hindered by the presence of 1-MT itself in the plant. Although the 1-MT levels detected in the plant did not seem to have any antimicrobial effect, the possibility of levels being high enough to interrupt, or at least affect flagellum formation, cannot be ruled out. By affecting flagellum formation, 1-MT directly affects the mobility of bacteria in the mesophyll and their capacity to reach the nutrients present in the apoplast. However, further metabolic and proteomic studies are needed to identify other compounds with antimicrobial activity that could help us to explain the reduced bacterial growth observed in the apoplast extracted from treated plants.

## Author Contributions

LS performed gene expression analysis. LS, EL, and BV set up the apoplast extraction method. EL conducted the heatmap visualization of the content of metabolites in the apoplast. AG-H, EL, and BV performed confocal analysis. AG-H carried out hormone analysis. MV performed sugar analysis. JG and GC set up amino acid method. BV and GC were involved in the design and discussion of the assays with support from PG-A. All authors contributed to the writing of the manuscript.

## Conflict of Interest Statement

The authors declare that the research was conducted in the absence of any commercial or financial relationships that could be construed as a potential conflict of interest.

## Footnotes

1 www.waters.com

2 http://www.omegabiotek.com

3 http://www.thermofisher.com

4 http://www.takarabio.com

5 http://www.qiagen.com
