# Supplementary material for: 1-Methyltryptophan Modifies Apoplast Content in Tomato Plants Improving Resistance Against Pseudomonas syringae
Source: Front Microbiol. 2018 Aug 31;9:2056. doi: 10.3389/fmicb.2018.02056 (PMC6127243; doi:10.3389/fmicb.2018.02056)
Supplement: Supplementary file 1 [file Table_1.DOCX]

**Supporting information**

**
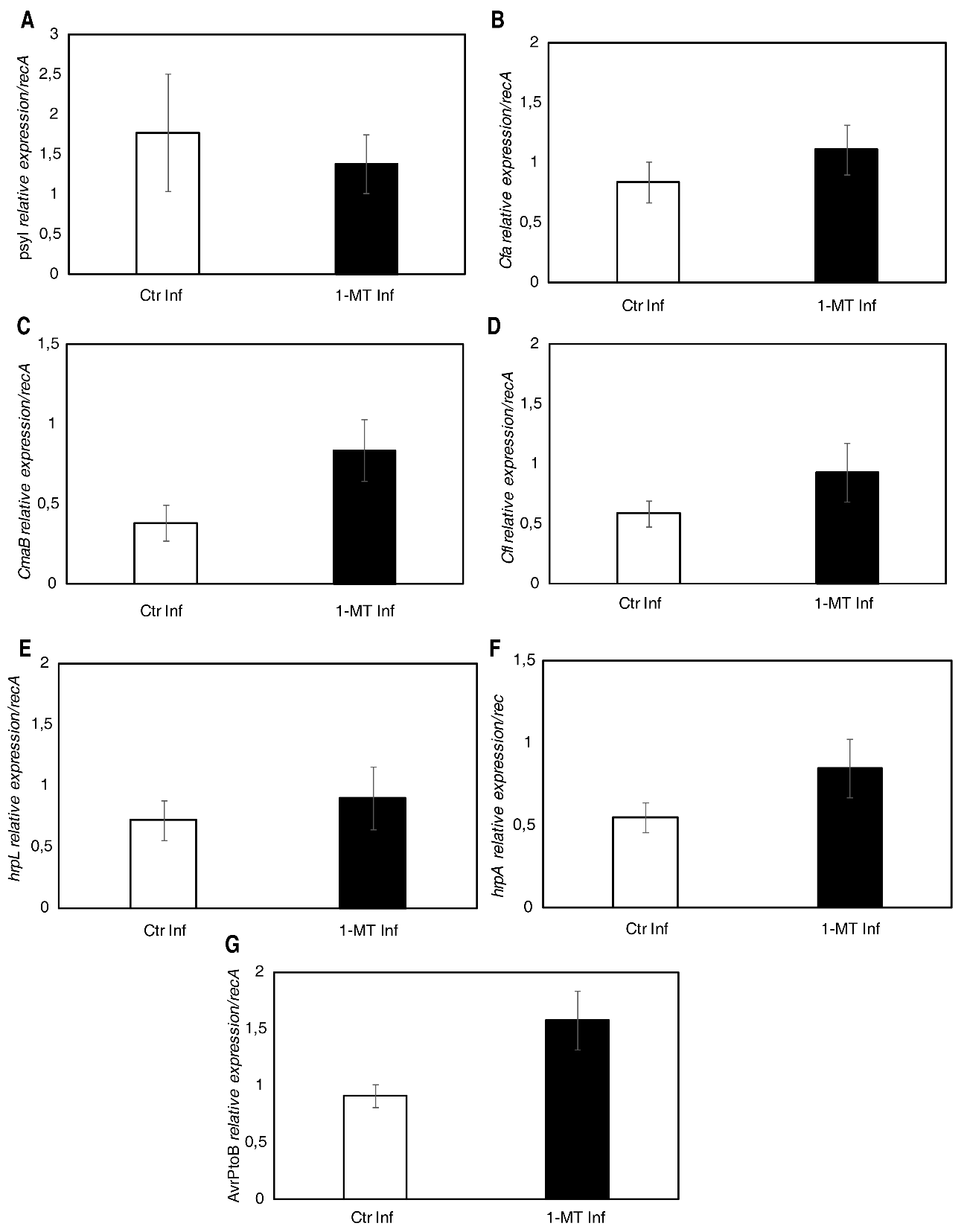
**

**Figure Supplementary 1**. Bacterial RNA extraction was performed from *P. syringae* extracted from infected leaves. Relative expression of following genes were analyzed during bacterial growth (A) Quorum sensing establishment-related genes (*psyI*), COR synthesis-related genes [(B) *cfa1*, (C) *cmaB* and (D) *cfl*], marker genes of the type III secretion system and the type III secretion system-associated pilus, respectively [(E) *hrpL* and (F) *hrpA*], (G) gene responsible for effector synthesis (*avrPtoB*). The recA gene was used as an endogenous reference gene.

**Table S1.** Amino acids, hormone and sugar content in apoplast extract.

The values are the average of three independent experiments ± SE. Apoplast amino acid and hormone concentration are expressed in ng mL^-1^ and sugar concentration is expressed in μg mL^-1^.

|  | Ctr | 1-MT | Ctr Inf | 1-MT Inf |
| --- | --- | --- | --- | --- |
| Thr | 406 ± 156 | 845 ± 244 | 235 ± 65 | 737 ± 247 |
| Val | 592 ± 156 | 559 ± 258 | 255 ± 96 | 496 ± 118 |
| Pro | 489 ± 47 | 1014 ± 39 | 495 ± 109 | 601 ± 92 |
| Ser | 59 ± 28 | 222 ± 81 | 20 ± 10 | 111 ± 26 |
| Ala | 271 ± 75 | 456 ± 142 | 417 ± 100 | 616 ± 156 |
| Trp | 180 ± 72 | 124 ± 59 | 164 ± 125 | 750 ± 335 |
| Phe | 225 ± 182 | 53 ± 15 | 38 ± 15 | 298 ± 259 |
| Glu | 2031 ± 736 | 3639 ± 887 | 2045 ± 327 | 5503 ± 1910 |
| Asp | 2054 ± 408 | 3590 ± 252 | 655 ± 185 | 2056 ± 667 |
| Tyr | 341 ± 112 | 511 ± 195 | 753 ± 102 | 1486 ± 291 |
| His | 103 ± 39 | 307 ± 22 | 964 ± 361 | 1154 ± 345 |
| Met | 35 ± 29 | 121 ± 29 | 76 ± 24 | 71 ± 12 |
| Gln | 478 ± 231 | 715 ± 137 | 722 ± 253 | 1385 ± 543 |
| Lys | 463 ± 254 | 687 ± 185 | 721 ± 273 | 1384 ± 632 |
| Pip | 230 ± 40 | 396 ± 42 | 1606 ± 223 | 2275 ± 970 |
|  | | | | |
| ABA | 38 ± 8 | 38 ± 3 | 48 ± 10 | 49 ± 4 |
| SA | 2 ± 2 | 8 ± 7 | 45 ± 27 | 19 ± 10 |
| OPDA | 27 ± 1 | 26,1 ± 0,1 | 42 ± 12 | 38 ± 7 |
| JA | 29 ± 5 | 42 ± 11 | 143 ± 42 | 127 ± 43 |
|  | | | | |
| Fructose | 50 ± 1 | 64,5 | 48 | 59 ± 11 |
| Glucose | 44 ± 7 | 40 ± 13 | n.d. | 37 |

**Table S2.** Primers used for bacterial gene expression analyses.

| Function | Gene | Primer |
| --- | --- | --- |
| Coronatine synthesis | *cfa1* | F 5’-AAAACCATCGTCGACATTCTG-3’  R 5’-GTTGGCGTTGAGGTCGATA-3’ |
|  | *cmaB* | F 5’-AATTCGACACCCGACAAGAC-3’  R 5’-ACTAGGGGCTTCAGGTCCAT-3’ |
|  | *cfl* | F 5’-ACAGCTGAAGCAGCACTTGA-3’  R 5’-CGAGGATCTCTCGGTAGTCG-3’ |
| Type III secretion system, type III secretion system-associated pilus and effector synthesis | *hrpL* | F 5’-TCTCCAGTGCGTGTTTCTTG-3’  R 5’-AGCTTTCCTGATACGGCTGA-3’ |
|  | *hrpA* | F 5’-CCTCCAAACTCACCAACCTT-3’  R 5’-CGGACTCTTTACTGGCCTTG-3’ |
|  | *avrPtoB* | F 5’-ACCCTATCGCGTCACAATTC-3’  R 5’-CATGAACGCCAGGTCCTTAT-3’ |
| Quorum sensing establishment | *psyI* | F 5’-GGCTTGAATGGAATGTTCGT-3’  R 5’-CAGGTGTTGATCAGCCGTAA-3’ |
| Flagellin synthesis | *fliC* | F 5’-ATCTGAACGGCAAGAACCTG-3’  R 5’-TGCGCTCAAAGTCAGAGAGA-3’ |
| Internal reference | *recA* | F 5’-CGGCAAGGGTATCTACCTCA-3’  R 5’-CTTTGCAGATTTCCGGGTTA-3’ |

**Table S3.** Primers used for plant gene expression analyses.

| Function | Gene | Primer |
| --- | --- | --- |
| abscisic stress-ripening protein 1 | *ASR1* | F 5’- ACACCACCACCACCTGT -3’  R 5’- GTGTTTGTGTGCATGTTGTGGA -3’ |
| pathogenesis-related protein 1 | *PR1* | F 5’- CCGTGCAATTGTGGGTGTC -3’  R 5’- GAGTTGCGCCAGACTACTTGAGT -3’ |
| pathogenesis-related protein 5 | *PR5* | F 5’- GAGGTTCATGCCAAACTGGTC -3’  R 5’- TCAACCAAAGAAATGTCC -3’ |
| allene oxide cyclase | *AOC* | F 5’- GCACGAAGAAGAGAAGAAAGGAGA -3’  R 5’- CGGTGACGGCTAGGTAAGTTT -3’ |
| elongation factor 1-alpha | *EF1α* | F 5’- GACAGGCGTTCAGGTAAGGA-3’  F 5’- GGGTATTCAGCAAAGGTCTC-3 |
